# γδ T Cell Immunotherapy—A Review

**DOI:** 10.3390/ph8010040

**Published:** 2015-02-12

**Authors:** Hirohito Kobayashi, Yoshimasa Tanaka

**Affiliations:** 1 Transfusion Medicine and Cell Processing, Department of Urology, Tokyo Women’s Medical University, 8-1 Kawada-cho, Shinjuku-ku, Tokyo 162-8666, Japan; 2 Center for Therapeutic Innovation, Graduate School of Biomedical Sciences, Nagasaki University, 1-14 Bunkyo-machi, Nagasaki 852-8521, Japan

**Keywords:** cancer immunotherapy, nitrogen-containing bisphosphonate, phosphoantigen, tumor, Vγ9Vδ2 T cell

## Abstract

Cancer immunotherapy utilizing Vγ9Vδ2 T cells has been developed over the past decade. A large number of clinical trials have been conducted on various types of solid tumors as well as hematological malignancies. Vγ9Vδ2 T cell-based immunotherapy can be classified into two categories based on the methods of activation and expansion of these cells. Although the *in vivo* expansion of Vγ9Vδ2 T cells by phosphoantigens or nitrogen-containing bisphosphonates (N-bis) has been translated to early-phase clinical trials, in which the safety of the treatment was confirmed, problems such as activation-induced Vγ9Vδ2 T cell anergy and a decrease in the number of peripheral blood Vγ9Vδ2 T cells after infusion of these stimulants have not yet been solved. In addition, it is difficult to *ex vivo* expand Vγ9Vδ2 T cells from advanced cancer patients with decreased initial numbers of peripheral blood Vγ9Vδ2 T cells. In this article, we review the clinical studies and reports targeting Vγ9Vδ2 T cells and discuss the development and improvement of Vγ9Vδ2 T cell-based cancer immunotherapy.

## 1. Introduction

T cells can be divided into two subsets—αβ T cells and γδ T cells—based on their expression of T cell antigen receptors (TCRs). The majority of αβ T cells recognize antigenic peptides in the context of MHC class I or class II and produce effector molecules that mediate the regulation and differentiation of other cells in the immune system. Although γδ T cells were discovered between 1984 and 1987 [1,2,3], relatively little is known about the types of antigens or the antigen presentation elements and pathways. In healthy adults, γδ T cells occupy 3 to 5% of peripheral blood T cells and play a role in the defense of the body against infections [4]. Typically, 50 to 75% of the peripheral blood γδ T cells express Vγ9 (also termed Vγ2) and Vδ2 from among the variable elements of TCR (Vγ9Vδ2 T cells) [4]. This subset of T cells can be readily activated in a TCR-dependent manner when exposed to small phosphorylated molecules such as isopentenyl diphosphate (IPP) and (*E*)-4-hydroxy-3-methylbut-2-enyl diphosphate (HMBPP) [5,6] or aminated alkyl molecules such as isobutylamine [7]. Although the precise mechanism has not yet been fully clarified, a consensus has been reached on the underlying roles of butyrophiline 3A1 (BTN3A1) in the Vγ9Vδ2 TCR recognition of phosphoantigens [8,9,10].

**Figure 1:**
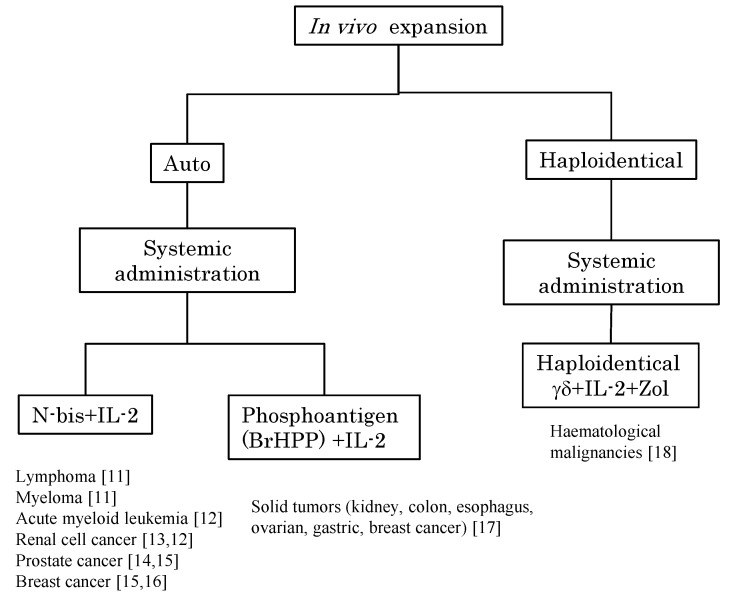
Peripheral blood Vγ9Vδ2 T cells can be stimulated by the systemic administration of phosphoantigen or N-bis and expanded by IL-2 for immunotherapy. The *in vivo* expansion of Vγ9Vδ2 T cells is divided into two strategies based on the cell origin, namely, autologous Vγ9Vδ2 T cells and haploidentical Vγ9Vδ2 T cells (the latter cells of which are derived from peripheral blood mononuclear cells of half-matched family donors). The stimulators were N-bis or phosphoantigen and all regimens involved the systemic administration of exogenous IL-2. Target tumor types and references [11,12,13,14,15,16,17,18] are indicated.

Conventional lymphokine-activated killer cells (LAKs) and CD3-activated T lymphocytes (CATs) utilized in adoptive immunotherapy for cancer contain not only NK cells and αβ T cells, but also γδ T cells. It is, however, difficult to demonstrate the roles of these cells clearly as it is very challenging to *ex vivo* expand these innate immune cells such as NK cells, dendritic cells, and the adaptive immune cells (e.g., antigenic peptide-specific αβ T cells) to a level where cancer immunotherapy is possible and efficacious. In stark contrast, Vγ9Vδ2 T cells proliferate vigorously *in vitro* in response to microbial and synthetic phosphoantigens [6]. In addition, it was demonstrated that synthetic nitrogen-containing bisphosphonates (N-bis), such as pamidronate (Pam) (used to treat hypercalcemia of malignancy), also stimulated human Vγ9Vδ2 T cells *in vitro* as well as *in vivo* [19]. As a result of these findings, cancer immunotherapy harnessing Vγ9Vδ2 T cells and synthetic phosphoantigens or N-bis has become possible and has been extensively developed.

**Figure 2:**
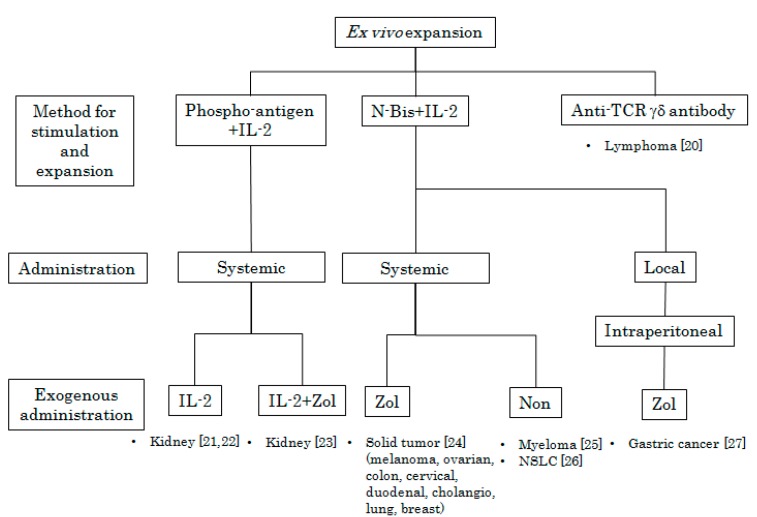
Peripheral blood mononuclear cells (PBMCs) were obtained from patients and treated with phosphoantigen or N-bis (specific stimulants for Vγ9Vδ2 T cells) in the presence of various concentrations of IL-2 *in vitro*. After incubation for appropriate periods, Vγ9Vδ2 T cells were intravenously or intraperitoneally administered following the systemic administration or local injection of IL-2, Zol, or IL-2 plus Zol. Target tumor types and references [20,21,22,23,24,25,26,27] are indicated.

Cancer immunotherapy utilizing Vγ9Vδ2 T cells can be classified into two categories based on the methods of activation and expansion of Vγ9Vδ2 T cells. The first is to stimulate Vγ9Vδ2 T cells *in vivo* by means of the systemic administration of phosphoantigens or N-bis (Figure 1). The second is to expand Vγ9Vδ2 T cells *ex vivo* using synthetic phosphoantigens or N-bis followed by the administration of cultured Vγ9Vδ2 T cells to the patient (Figure 2). These therapeutic interventions can be undertaken in combination with cytokines such as interleukin-2 (IL-2) and/or chemotherapeutic agents.

## 2.* In Vivo* Stimulation of Vγ9Vδ2 T Cells Using Synthetic Antigens and IL-2

Kunzmann *et al.* initially reported that Pam could stimulate Vγ9Vδ2 T cells in the peripheral blood [19]. In their trial, four of ten patients had acute-phase reactions (APRs; fever and influenza-like symptoms) after Pam treatment and all four of these patients had a substantial increase in the proportion of Vγ9Vδ2 T cells. Rossini *et al.* reported that 42% of patients (17 of 40) undergoing infusion of zoledronic acid (Zol), one of the strongest N-bis that is widely used in clinics for metastatic bone tumors, experienced APRs. Based on the receiver operating characteristic (ROC) curve, they concluded that having more than 25 γδ T cells/μL (*p* = 0.032) or 3.0% γδ T cells (*p* = 0.027) were risk factors of APR [28]. Proliferative responses of Vγ9Vδ2 T cells to N-bis are dependent on IL-2 [29]. Stimulated Vγ9Vδ2 T cells produce cytokines such as interferon-γ (IFN-γ) and tumor necrosis factor-α (TNF-α) and exhibit specific cytotoxicity against various tumor cells, including lymphoma and myeloma cell lines [30]. Wilhelm and coworkers first demonstrated that *in vivo* Vγ9Vδ2 T cell stimulation by Pam and low-dose IL-2 was safe and could induce objective tumor responses in patients with low-grade non-Hodgkin lymphoma (NHL, *n* = 11) and multiple myeloma (MM, *n* = 8) [11]. It was noted that patient selection was a prerequisite for successful treatment (namely, positive *in vitro* responses of Vγ9Vδ2 T cells to Pam and IL-2). In addition, the dose and timing of IL-2 administration is important. In this report, patients who showed positive *in vitro* responses to Pam plus IL-2 achieved objective clinical responses, and patients who received IL-2 at dose levels of 1 × 10^6^ to 2 × 10^6^ IU from day 1 to day 6 after Pam infusion (90 mg) responded to the treatment. Ten patients who received IL-2 from day 3 through day 8 after an initial Pam infusion (90 mg), however, did not achieve an objective tumor response. This may indicate that IL-2 administration three days after Pam infusion was too late to avoid activation-induced cell death of Vγ9Vδ2 T cells.

Dieli and coworkers conducted a phase I clinical trial to determine the anti-tumor effect of the single or combined administration of Zol and IL-2 in patients with metastatic castration-resistant prostate cancer (CRPC) [14]. In this study, 18 patients were enrolled and divided into two groups. One group (*n* = 9, Cohort A) received 4 mg of Zol alone, and the other (*n* = 9, Cohort B) received 4 mg of Zol and 6 × 10^5^ IU of IL-2 via subcutaneous injection at the same time. The treatments were performed every 21 days and immune-monitoring was performed at 3, 6, and 12 months. Six of nine patients in Cohort B, but only two of nine patients in Cohort A, showed increased numbers of peripheral blood Vγ9Vδ2 T cells with an effector-memory phenotype. The number of peripheral blood Vγ9Vδ2 T cells was evaluated 3 months after the initial treatment in five patients in Cohort A; four patients died due to CRPC within 3 months. Four of five evaluable patients in Cohort A showed markedly decreased numbers of peripheral blood Vγ9Vδ2 T cells after the therapy and the remaining patient showed slightly decreased numbers. It is of note that almost all peripheral blood Vγ9Vδ2 T cells disappeared in four patients in Cohort A after the fourth administration of Zol (3 months after the first administration). This phenomenon indicates the importance of the administration of exogenous IL-2 to maintain peripheral blood Vγ9Vδ2 T cells. Serum TNF-related apoptosis-inducing ligand (TRAIL) levels were maintained in patients with Zol + IL-2, and higher TRAIL levels were associated with favorable clinical outcomes. Serum prostate specific antigen (PSA) levels were inversely correlated with the total Vγ9Vδ2 T cell numbers and effector memory-type Vγ9Vδ2 T cell numbers.

IL-2 therapy is a standard treatment for metastatic renal cell carcinoma (RCC) in the National Comprehensive Cancer Network (NCCN) guidelines (ver. 2, 2014). Because the side effects of high-dose IL-2 therapy (6 × 10^5^ IU/kg/8 h, for example) are very serious and intensive care unit–based management is required, its availability is limited. For example, only low-dose IL-2 therapy (0.35–2.1 × 10^6^ IU/body) is approved in Japan because of toxicity. Administration of Zol plus low-dose IL-2 seems to be a reasonable and attractive therapy for metastatic RCC. Lang *et al.* reported a pilot trial of Zol and two different doses of IL-2 (higher: 7 × 10^6^ IU/m^2^/day and lower: 1 × 10^6^ IU/m^2^/day) administration for patients with metastatic RCC and attempted to determine the optimal dose of Zol and IL-2 for the activation and expansion of peripheral blood Vγ9Vδ2 T cells, which might correlate with favorable clinical outcomes [13]. Twelve patients were enrolled in this study and six received 3 consecutive weekly cycles of 4 mg of Zol followed by 7 × 10^6^ IU/m^2^/day of IL-2 for 5 consecutive days. Three patients received 4 mg of Zol and 1 × 10^6^ IU/m^2^/day of IL-2 for 5 consecutive days. None of the patients who received 4 mg of Zol plus 7 × 10^6^ IU/m^2^/day of IL-2 showed an increased number of peripheral blood Vγ9Vδ2 T cells, and one of three patients who received 4 mg of Zol and 1 × 10^6^ IU /m^2^/day of IL-2 had an increased number of peripheral blood Vγ9Vδ2 T cells. The authors considered 4 mg of Zol to be too strong as a stimulant for Vγ9Vδ2 T cells to be combined with a higher dose of IL-2. To determine the optimal dose of Zol, three other patients received decreased doses (0.4–3.0 mg) of Zol and 1 × 10^6^ IU/m^2^/day of IL-2 for 5 consecutive days. All three patients who received decreased doses (0.4–3.0 mg) of Zol and 1 × 10^6^ IU/m^2^/day of IL-2 exhibited an increased number of Vγ9Vδ2 T cells in the peripheral blood and displayed stable disease. The most common adverse events were the same as those reported in IL-2 monotherapy; thus, the regimens were well tolerated. Intravenous IL-2 administration (every 8 h at 7.2 × 10^4^ IU/kg up to 15 doses) for patients with nasopharyngeal carcinoma induced increased levels of serum TNF-α, IL-6, soluble IL-2 receptor, IL-10, and soluble CD8. These soluble factors might influence the maintenance of Vγ9Vδ2 T cell responses.

The same research group also conducted a phase I trial in which Zol and low-dose IL-2 were administered to 10 advanced metastatic breast cancer patients [16]. The patients received 4 mg of Zol, followed by 1 × 10^6^ IU/body of IL-2 every 21 days. The regimen was well tolerated and Vγ9Vδ2 T cells exhibited an effector phenotype in all patients. The robust and sustained expansion of peripheral blood Vγ9Vδ2 T cells was observed in three out of ten patients (one of which had PR and two of which had SD).

Bromohydrin diphosphate (BrHPP, IPH101, Phosphostim) is another potent stimulator of human Vγ9Vδ2 T cells *in vitro* and *in vivo* [31]. Bennouna and coworkers conducted a phase I trial to determine the maximum tolerated dose (MTD) and safety of BrHPP plus low-dose IL-2 in patients with solid tumors [17]. In this study, 200 to 1,800 mg of BrHPP with or without 1 × 10^6^ IU of IL-2 was subcutaneously administrated to 28 patients on day 1 through day 7 every 3 weeks. The regimen of 1500 mg of BrHPP with or without IL-2 was well tolerated and the expansion of peripheral blood Vγ9Vδ2 T cells was dependent on IL-2. Phosphoantigen-specific proliferation of Vγ9Vδ2 T cells diminished after repetitive administrations of BrHPP plus IL-2 and no objective response was observed in 28 patients. These clinical trial results are summarized in Table 1.

**Table 1:** *In vivo* stimulation of Vγ9Vδ2 T cells using synthetic antigens and IL-2.

Reference	Number of Patients	Disease	Stimulant		Exogenous IL-2		Cycle	Numbers of Cycles	Clinical Outcome
			Dose	Schedule	Dose (IU), Route, Patients	Schedule			
Wilhelm *et al.*, **2003** *Blood* [11]	10	MM: 4	Pam (90 mg)	d1	0.25–3 × 10^6^, iv	d3–d8	at least 3 weeks	1–6	PD: 8, SD: 1, NE: 1
		CLL: 4							
		IC: 1							
		MZL: 1							
	9	MM: 4			0.25 × 10^6^, iv, *n* = 3, 0.5–2 × 10^6^, iv, *n* = 3, 1–2 × 10^6^, iv, *n* = 3	d1–6		1–2	PD: 3
		FCL: 4						1–9	PD: 1, SD: 2, PR: 1
		MZL: 1						4–8	SD: 1, PR: 2
Dieli *et al.*, **2007** *Cancer Res.* [14]	9	HRPC	Zol (4 mg)	d1	No		21 days	2–17 (at 12 months)	SD: 1, PR: 1, PD: 1, death: 6
	9				0.6 × 10^6^, sc	d1		7–17 (at 12 months)	SD: 4, PR: 2, PD: 1, death: 2
Lang *et al.*, **2011** *Cancer Immunol. Immunother.* [13]	6	RCC	Zol (4 mg)	d1, d8, d15	7 × 10^6^ U/m^2^/day, sc	d1–5, d8–12, d15–19	28 days	<1: *n* = 2,	SD:3, PD:1, NA: 3
								2–10: *n* = 4,	
								NA: *n* = 1	
	3				1–2 × 10^6^ U/m^2^/day, sc			<1	NA:1, SD:2
								3	
								33	
	3		Zol (0.4–3.0 mg)		1–2 × 10^6^ U/m^2^/day, sc			4	SD: 3
								7	
								16	
Meraviglia *et al.*, **2010** *Clin. Exp. Immunol.* [16]	10	Breast cancer	Zol (4 mg)	d1	1 × 10^6^ U/m^2^/day, sc	d1	21 days	<4: *n* = 2	PD: 2, SD: 2, PR: 1 at 9 months
								5–13: *n* = 3	
								14–18: n = 1	PD: 1, SD: 2, PR: 1 at 12 months
								18<, *n* = 4	
Bennouna *et al.*, **2010** *Cancer Immunol. Immunother.* [17]	28	RCC: 18	BrHPP (200 mg/m^2^)	d1	1 × 10^6^ U/m^2^/day, sc, from 2 cycles	d1–7	21 days	18	SD: 12, PD: 16 at 3 cycles
		Colon Ca: 3	(600 mg/m^2^)						
		Esophagus Ca: 3	(1200 mg/m^2^)					10	
		Gastric Ca: 1	(1500 mg/m^2^)					26	
		Ovarian Ca: 1	(1500 mg/m^2^)		1 × 10^6^ U/m^2^/day, sc, from 1 cycle			39	
		Breast Ca: 2	(1800 mg/m^2^)		1 × 10^6^ U/m^2^/day, sc, from 2 cycles			16 cycles	

Abbreviations: MM: multiple myeloma, CLL: chronic lymphocytic leukemia, IC: Immunocytoma, MZL: mantle zone lymphoma, FCL: follicular cell lymphoma, HRPC: hormone-refractory prostate cancer, RCC: renal cell carcinoma, Pam: pamidronate, Zol: zoledronic acid, iv: intravenous, sc: subcutaneous.

### 2.1. In Vivo Activation of γδ T Cells After Adoptive Cell Therapy

The *in vivo* activation of γδ T cells in patients with advanced hematological malignancies was reported by Wilhelm and coworkers [18]. Four patients who had undergone prior immunosuppressive chemotherapy with Hi-Cy/Flu (fludarabine 20–25 mg/m^2^ day -6 through day -2 and cyclophosphamide 30–60 mg/kg day -6 and day -5) received CD4/8 T cell-depleted luekapheresis products from one haploidentical donor. All patients then received 4 mg of intravenous Zol on day 0 and 1 × 10^6^ IU /m^2^ of IL-2 on day +1 through day +6. This resulted in a marked *in vivo* expansion of donor γδ T cells. Although all patients were refractory to prior therapies, three out of four achieved complete remission without graft-versus-host disease (GVHD).

### 2.2. Theoretical Concern and Appropriate Dosages of N-bis/IL-2

*In vivo* Vγ9Vδ2 T cell expansion regimens using N-bis (Zol or Pam) or BrHPP followed by low-dose IL-2 were well tolerated. Although the administration of IL-2 was essential for the activation, a high dose of IL-2 was not necessary to sustain a high Vγ9Vδ2 T cell level. Based on these reports, it is likely that the safety range of IL-2 administration is between 1 × 10^6^ IU/body/day and 1 × 10^6^ IU/m^2^/day for up to 7 consecutive days. Although 1 μM (= 290.1 ng/mL) to 5 μM of Zol concentration is enough to activate and expand Vγ9Vδ2 T cells *in vitro*, the optimal dose of Zol for *in vivo* activation has not yet been determined [24,25,26,32]. When Zol was administered at a single dosage of 4 mg to ten Japanese patients with metastatic bone tumors, the peak serum concentration (Cmax) was 426 ± 101 ng/mL and the area under the curve (AUC) 0 to 24 h was 576 ± 130 ng/h/mL (public data from Novartis Pharma). Based on these data, a lower dose of Zol seems to be enough to activate peripheral blood Vγ9Vδ2 T cells, because of the efficient uptake of Zol by monocyte-lineage cells via fluid phase endocytosis [33]. In fact, Lang and coworkers reported that 0.4 mg of Zol plus 1 × 10^6^ IU/m^2^ of IL-2 induced the expansion of peripheral blood Vγ9Vδ2 T cells [13]. Although the administration of Zol or BrHPP plus low-dose IL-2 increases the proportion of Vγ9Vδ2 T cells in an effector/memory (E/M) state, the treatments often reduce the total number of Vγ9Vδ2 T cells and induce anergy in the T cell subset.

Miyagawa *et al.* demonstrated that the activation of primary Vγ9Vδ2 T cells by Pam was dependent on the presence of monocyte-lineage cells [33]. When Zol is internalized into the cytoplasm of the monocyte-lineage cells, it inhibits the rate-limiting enzyme farnesyl diphosphate synthase (FPPS) in the mevalonate pathway, thereby increasing the level of the upstream metabolite IPP. The higher level of IPP is sensed by the intracellular domain of BTN3A1, which may induce an extracellular conformational change or oligomerization. Finally, the structural change in BTN3A1 by IPP may be recognized by Vγ9Vδ2 TCR [9,10].

The mechanisms underlying the activation-induced Vγ9Vδ2 T cell anergy and the decrease in the number of peripheral blood Vγ9Vδ2 T cells after infusion of N-bis have not yet been fully delineated. In patients naïve to N-bis, a significant reduction in both total lymphocytes and Vγ9Vδ2 T cell subset was observed 2 days after Zol infusion. Although all lymphocyte counts other than Vγ9Vδ2 T cells returned to baseline one year after infusion, Vγ9Vδ2 T cells remained significantly lower in both proportion and absolute numbers [34]. Activation-induced Vγ9Vδ2 T cell anergy might be triggered by the involvement of nonprofessional antigen-presenting cells that fail to express costimulatory molecules. The tachyphylaxis is observed in both Zol plus IL-2 and BrHPP + IL-2 regimens.

It is noteworthy that the Zol plus IL-2 regimen induces the upregulation of serum vascular endothelial growth factor (VEGF) levels in patients with both solid and hematopoietic tumors, resulting in the promotion of tumor angiogenesis. Kunzmann and coworkers conducted a phase I/II trial of the Zol plus IL-2 regimen for patients with RCC, melanoma, and acute myeloid leukemia (AML) [12]. Twenty-one patients were enrolled in this study and a total of 58 treatment cycles were performed. The regimen was safe and well tolerated. Although no objective response was observed in RCC patients, two AML patients had PR. It is likely that an increased level of serum VEGF is correlated with a lack of clinical responses, especially in angiogenesis-dependent RCC.

It is suggested that inhibition of the mevalonate pathway results in a depletion of geranylgeranyl diphosphate, leading to the activation of caspase I, which in turn maturates IL-18. IL-18 mediates the differentiation and activation of helper NK cells, which promote the expansion of Vγ9Vδ2 T cells [35,36,37]. IL-21 inhibits the division of Vγ9Vδ2 T cells stimulated by phosphoantigens, but enhances cytolytic activities [38]. IL-18 and IL-21 were examined in a clinical trial for patients with NHL and metastatic melanoma [39,40]. These *in vitro* studies and clinical trials will give us clues on how to overcome treatment-induced unfavorable reactions such as the induction of VEGF in immunotherapy using Vγ9Vδ2 T cells.

## 3. Adoptive Immunotherapy Using *Ex Vivo* Activated Vγ9Vδ2 T Cells

Several clinical trials using *ex vivo* expanded γδ T cells have been conducted in which various culture conditions were tested, such as various concentrations of stimulants and IL-2. Various tumors have been targeted, including hematological malignancies, RCC, non-small-cell lung carcinoma (NSCLC), malignant melanoma (ML), ovarian carcinoma (OC), and breast cancer (BC). In addition to Vγ9Vδ2 T cell infusion, additional treatments, such as the systemic administration of IL-2 or Zol, have been examined in clinical trials. These clinical trial results are summarized in Table 2 and Table 3.

### 3.1. Role of Vδ1 T Cells

Anti-TCR γδ monoclonal antibody (mAb) stimulated both Vδ1 and Vδ2 T cells. The Vδ1 T cells also exhibited potent cytotoxic activity against tumor cells *in vitro*, equivalent to that of Vδ2 T cells [20]. The anti-tumor activity of Vδ1 T cells is, however, controversial. Peng *et al.* reported that a dominant Vδ1 T cell population among lymphocytes infiltrating breast tumors exhibited a potent ability to suppress naïve and effector T cell responses and to block the maturation and function of dendritic cells (DCs) [41]. In contrast, *ex vivo* expanded Vδ1 T cells using Vδ1 mAb and IL-2 killed glioblastoma multiform (GBM) cell lines and primary tumor-derived GBM cells [42]. It is likely that Vδ1 T cells can be further divided into two subsets, cytotoxic Vδ1 cells and regulatory Vδ1 cells. The former subset may exist in lymphocyte cultures stimulated with anti-Vδ1 mAb and IL-2, and the latter in tumor-infiltrating lymphocytes, the peripheral blood of children who underwent liver transplantation and exhibited allograft tolerance, and lymphocytes found in the uteri of pregnant women.

**Table 2:** *Ex vivo* expansion conditions for adoptive immunotherapy using *ex vivo* activated Vγ9Vδ2 T cells.

Reference	Number of Patients	Disease	Cell Source	Culture condition				
				IL-2	Stimulant (Concentration)		Serum	Culture days
Kobayashi *et al.*, **2007** *Cancer Immunol. Immunother.* [22]	7	RCC	PB	100 U/mL	2M3B1-PP	100 μM	2% Auto serum	14 days
Bennouna *et al.*, **2008** *Cancer Immunol. Immunother.* [21]	10	RCC	Leukapheresis	328 U/mL: d1, 984 U/mL: d4–14	BrHPP	3 μM	9% FCS	14 days
Kobayashi *et al.*, **2011** *Cancer Immunol. Immunother*. [23]	11	RCC	Leukapheresis (1 leukapheresis for 2 treatments)	100 U/mL	2M3B1-PP	100 μM	2% Auto serum	11 days
Nicol *et al.*, **2011** *Br. J. Cancer* [24]	18	Melanoma: 4, Ovarian cancer: 1, Colon cancer: 1	Luekapheresis 1 leukapheresis for 8 treatments	700 IU/mL, d0; 350 IU/mL, every 2–3 days	Zol	1 μM	10% AB serum	7–14 days
		Melanoma: 3						
		Adenocarcinoma: 1						
		Cholangiocarcinoma: 1						
		Ovarian carcinoma: 1						
		Colon cancer: 2						
		Duodenal cancer: 1						
		Breast cancer: 2, Cervical cancer: 1						
Nakajima *et al.*, **2010** *Eur. J. Cardiothorac. Surg.* [26]	10	Non-small-cell lung cancer	Peripheral blood 70 mL	1000 U/mL	Zol	5 μM	10% Auto serum	14 days
Abe *et al.*, **2009** *Exp. Hematol.* [25]	6	Multiple myeloma	Peripheral blood	1000 U/mL	Zol	5 μM	Auto serum	14 days
Wada *et al.*, **2014** *Cancer Med.* [27]	7	Gastric cancer	Leukapheresis	1000 U/mL	Zol	5 μM	Auto serum	14 days

**Table 3:** Adoptive immunotherapy using* ex vivo* activated Vγ9Vδ2 T cells.

References	No. of Transferred γδ T Cells		Cycle		Route	Exogenous Administration			Clinical Outcome
	Each Cycle	Total	Interval	Cycles		Zol	IL-2: Dose/Schedule		
Kobayashi *et al.*, **2007** *Cancer Immunol. Immunother.* [22]	5 × 10^6^ to 3.57 × 10^9^	0.1–40.8 × 10^9^	Weekly	6–12	iv	No	7 × 10^5^ IU, iv	d1	
Bennouna *et al.*, **2008** *Cancer Immunol. Immunother.* [21]	1 × 10^9^, *n* = 1	1.45–18.3 × 10^9^	3 weeks	3	iv	No	Cycle 1: no, cycle 2: 2 × 10^6^ IU/m^2^, sc	d1–d7	SD: 6, PD: 4
	4 × 10^9^, *n* = 6			16					
	8 × 10^9^, *n* = 3			8					
Kobayashi *et al.*, **2011** *Cancer Immunol. Immunother.* [23]	9.4 × 10^6^–24.0 × 10^9^	1.5×10^9^–46.7 × 10^9^	3 weeks	Average 4.2	iv	4 mg, iv. d1	1.4 × 10^6^ IU, iv	d1–5	SD: 5, PD: 5, CR: 1
	mean: 1.4 × 10^9^	mean: 22.0 × 10^9^							
Nicol *et al.*, **2011** *Br. J. Cancer* [24]	0.04–2.8 × 10^9^	0.1–5.5 × 10^9^		Up to 8	iv	1 mg, iv, 24 h before cell transfer; 1 mg, iv, just before cell transfer	No	No	SD: 2, PD: 4
	0.3–2.2 × 10^9^	1.0–7.2 × 10^9^							SD: 1, PD: 7, NE: 1
	0.3–1.9 × 10^9^	0.9–4.0 × 10^9^							PR: 2, CR: 1
Nakajima *et al.*, **2010** *Eur. J. Cardiothorac. Surg.* [26]		2.6–31.4 × 10^9^	2 weeks	3–12 (mean 6)	iv	No	No		SD: 3, PD: 5 NE: 2
Abe *et al.*, **2009** *Exp. Hematol.* [25]	0.07–5.2 × 10^9^	3.0–20.0 × 10^9^	2 weeks	4–8 (mean 7)	iv	No	No		Decreased M-protein levels: 4
Wada *et al.*, **2014** *Cancer Med* [27]	0.06–6.49 × 10^9^	0.06–25.0 × 10^9^	weekly	1–4 (mean 3)	ip	1 mg, iv at 1st therapy, 1 mg intraperitoneal injection, after 2nd therapy	No		Ascites, disappeared: 1, Reduced: 1, No change: 5

### 3.2. Clinical Trials Using Ex Vivo Activated Vγ9Vδ2 T Cells

Most groups used pyrophosphomonoester antigens or Zol to specifically stimulate Vγ9Vδ2 T cells. For example, in 1996 we designed and synthesized 2-methyl-3-butenyl-1-diphosphate (2M3B1PP), a pyrophosphomonoester antigen, which was approximately tenfold more potent than IPP in stimulating Vγ9Vδ2 T cells [43].

We first conducted a pilot study of adoptive immunotherapy using 2M3B1PP-stimulated autologous Vγ9Vδ2 T cells and low-dose IL-2 against advanced RCC patients [22]. Seven patients were enrolled and received 5 × 10^6^ to 3.57 × 10^9^ of Vγ9Vδ2 T cells every week for 6 to 12 weeks. They also received an intravenous infusion of 7 × 10^5^ IU of teceleukin (recombinant human IL-2, Shionogi & Co., Ltd., Osaka, Japan) after the adoptive transfer of Vγ9Vδ2 T cells. Adverse events were graded using the National Cancer Institute Common Toxicity Criteria Version 3.0 (CTCAE, Ver.3.0). All patients had IL-2-related adverse events such as fever, fatigue, elevation of liver transaminase, and eosinophilia, all of which were graded as 1 or 2. In immunological monitoring, five of seven patients showed an increased number of peripheral blood Vγ9Vδ2 T cells. We also evaluated the anti-tumor effects by assessing tumor-volume-doubling time (DT) on serial computed tomography (CT). DT was calculated in five patients, three of whom showed prolongation of DT. Although the clinical benefits were considered moderate, the regimen was well tolerated. Whereas six of seven patients died, one patient (Number 5 in Table 1 of reference 39) survived for over ten years after the treatment. He received 12 infusions, in which 1.11 × 10^9^ of Vγ9Vδ2 T cells were administered on average during the clinical trial. He received an additional 34 Vγ9Vδ2 T cell adoptive transfers every month from the start of the clinical trial until the anti-angiogenesis drug (Sorafenib) became available in Japan in May 2008, when he started receiving sequential therapy of tyrosine kinase inhibitors and mammalian target of rapamycin (mTOR) inhibitor (Everolimus).

Bennouna and coworkers conducted a phase I clinical trial of adoptive immunotherapy using Vγ9Vδ2 T cells stimulated by BrHPP and expanded with IL-2 in patients with metastatic RCC (21). The aim of this clinical study was to determine the MTD and safety of Innacell γδ™, which contains autologous Vγ9Vδ2 T cells expanded by BrHPP plus IL-2. Ten patients were enrolled in this trial and received Innacell γδ™ in the first cycle and then Innacell γδ™ + low-dose IL-2 (2 × 10^6^ IU/m^2^ from day 1 through day 7) from the second cycle at 3 week intervals. A dose-escalation study for Innacell γδ™ was conducted to evaluate the safety and tolerability of the treatment. Patients received 1 × 10^9^ to 8 × 10^9^ of Innacell γδ™. One patient who received 8 × 10^9^ of Innacell γδ™ was diagnosed with disseminated intravascular coagulation by laboratory data at the second cycle, though no clinical symptoms were reported. Other adverse events were considered to be IL-2-related. The results demonstrated that adoptive immunotherapy using Vγ9Vδ2 T cells plus low-dose IL-2 was well tolerated.

We also conducted a phase I/II trial of the adoptive transfer of 2M3B1PP-stimulated Vγ9Vδ2 T cells in combination with Zol and IL-2 in patients with advanced RCC [23]. It is noteworthy that Vγ9Vδ2 T cells exert specific cytolysis in a TCR-dependent manner when they encounter human tumor cells pulsed with N-bis, such as Pam and Zol [33,44]. It was demonstrated that the inhibition of FPPS by N-bis results in the accumulation of IPP in tumor cells, leading to the activation of Vγ9Vδ2 T cells [45]. We employed a strategy consisting of N-bis infusion to sensitize tumor cells, followed by the adoptive transfer of 2M3B1PP-stimulated Vγ9Vδ2 T cells and low-dose IL-2 [23]. Although 1 μM (= 290.1 ng/mL) to 5 μM of Zol concentration was sufficient to activate and expand Vγ9Vδ2 T cells *in vitro*, 10 μM of Zol concentration was needed to inhibit the prenylation of proteins in osteoclast cells and resulted in the accumulation of internal IPPs (public data from Novartis Pharma). We administered 4 mg of Zol to achieve sufficient concentrations in tumor cells. It is difficult to prove the hypothesis in humans, but a report of an animal model by Benzaïd *et al.* strongly supported it [46]. Vγ9Vδ2 T cells infiltrated the tumor and inhibited the growth of tumor cells producing a high level of IPP, but not those with a low level of IPP in Zol-treated mice with subcutaneous breast cancer xenografts. It was also suggested that IPP secreted into the extracellular matrix promoted Vγ9Vδ2 T cell chemotaxis to the tumor [46]. Eleven RCC patients with lung metastasis refractory to IFN-α therapy were enrolled and received 4 mg of Zol intravenously, followed by 1.4 × 10^6^ IU of teceleukin from day 0 through day 4. They received up to six adoptive transfers of 2M3B1PP-stimulated autologous Vγ9Vδ2 T cells and IL-2 infusions following Zol administration. The treatment was given to patients every 3 weeks. We cultured Vγ9Vδ2 T cells in the presence of 2M3B1PP and IL-2 for 11 days before infusion, because the level of IL-2 receptor α (CD25) expression on 2M3B1PP-stimulated Vγ9Vδ2 T cells was highest around day 11 and the CD25 expression decreased thereafter to a baseline level by day 14. Although the time course of CD25 expression varied from individual to individual, we did not extend the culture duration more than 11 days, because the CD25 expression was essential for the *in vivo* expansion of Vγ9Vδ2 T cells by infusion of low-dose IL-2. We were also concerned that high-dose IL-2 might induce too much proliferation, resulting in a reduction of telomere length and limited proliferation *in vivo*. In order to maintain the preferable cellular functions of Vγ9Vδ2 T cells, we therefore administered IL-2 to patients for only five days after the transfer. Low-dose IL-2 may increase the frequency of regulatory T cells (Tregs), which may inhibit the cytotoxic activity of Vγ9Vδ2 T cells according to *in vitro* studies [47]. Koreth and coworkers reported that the daily administration of low-dose IL-2 induced the selective expansion of functional Tregs, resulting in clinical improvement of chronic GVHD [48]. In this study, in order to control GVHD, low-dose IL-2 was administered daily for 8 weeks. On the other hand, phosphoantigen-activated Vγ9Vδ2 T cells could down-modulate IL-2-induced expansion of Tregs [49]. In our study [23], eleven patients with lung metastasis of RCC refractory to IFN-α were treated with low-dose IL-2 for 5 consecutive days every 3 weeks, and clinical responses were evaluated by CT examination using the Response Evaluation Criteria in Solid Tumors (RECIST). Five patients showed PD, five patients SD, and one patient CR [50]. Two patients died during the trial and four others died of RCC after the study (11.5, 27, 30, and 58 months after enrollment). One patient received best supportive care and another three patients received anti-angiogenetic drugs (sorafenib, sunitinib, and sequential administration of sorafenib/sunitinib). The remaining five patients survived an average of 93 months following treatment. Four of five patients received anti-angiogenetic drugs (sequential combinations of sorafenib, sunitinib, and axtinib). One patient who had shown complete remission after six cycles of phosphoantigen-activated Vγ9Vδ2 T cells, IL-2, and Zol administration maintained remission for 90 months after the sixth treatment without further treatment. The regimen was well tolerated and most adverse events were IL-2-related. A characteristic adverse event was lymphopenia, which was prominent within a day after treatment and the symptoms of which disappeared the next day without any treatment. In our protocol, a decreased number and frequency of peripheral blood Vγ9Vδ2 T cells were observed as the cycle of treatments progressed. In addition, a lower response rate compared with that in the first treatment of peripheral blood Vγ9Vδ2 T cells to phosphoantigens was seen—This was also observed in other clinical trials of Zol and IL-2 administration. The precise mechanism of this functional anergy remains unknown. We have not examined the frequency of Tregs before and after treatment, and further investigations are thus necessary.

Nicol and coworkers performed a clinical study to evaluate the safety and feasibility of the adoptive transfer of large numbers of *ex vivo* expanded autologous Vγ9Vδ2 T cells using Zol and IL-2 in combination with Zol infusion to patients with advanced solid tumors [24]. Eighteen patients were enrolled, six of whom were treated on a dose-escalation protocol. The maximum dose achieved in six patients was 2.8 × 10^9^ cells, and no transferred cell-related adverse events were observed. Zol was first administered to patients (1 mg per treatment); 24 h later, the patients were again infused with 1 mg of Zol immediately before cell transfer. Of the fifteen evaluable patients, three patients had SD and twelve had PD during the study period. Three patients (two breast cancer and one cervical cancer; two of whom showed PR and one CR) were treated concurrently with other therapies (two with chemotherapy and one with hormone therapy). The researchers also demonstrated the migration of infused *ex vivo* expanded Vγ9Vδ2 T cells labeled with radioactive ^111^indium oxine (^111^In) in three patients. The cells infiltrated the lungs and remained there for 4 to 7 h. Then, cell numbers (estimated based on γ-ray radioactivity) slowly decreased with gradual migration into the liver and spleen. In one patient with a large metastatic mass of 84 mm in diameter in the left adrenal gland, the cells accumulated in the metastatic site 1 h after infusion and maximal activity was seen in the tumor area at 4 to 48 h. Because the patients were not infused with IL-2 after the transfer, the toxicity profile of Vγ9Vδ2 T cells plus Zol was not evaluated. The cessation of exposure to a high concentration of IL-2 (700 or 350 IU/mL) in culture may limit the survival and function of Vγ9Vδ2 T cells after cell transfer.

Adoptive immunotherapy using autologous Vγ9Vδ2 T cells to patients with advanced and/or recurrent NSCLC was reported from a Japanese group [26,51]. Nakajima and coworkers conducted a phase I clinical trial using Zol-stimulated (5 μM), IL-2-expanded (1,000 IU/mL) Vγ9Vδ2 T cells against recurrent NSCLC [26]. Ten patients were enrolled and received 3 to 12 autologous Vγ9Vδ2 T cell administration every other week. The number of Vγ9Vδ2 T cells administered ranged from 2.6 to 14.5 × 10^9^ cells. The treatment was safe and well tolerated. Three patients showed SD and 5 showed PD four weeks after the last treatment. The SD patients received more than 10 × 10^9^ cells, and the elevation of plasma IFN-γ concentration was observed.

Abe *et al.* conducted a pilot study using Zol-stimulated (5 μM) and IL-2-expanded (1,000 IU/mL) Vγ9Vδ2 T cells in patients with MM [25]. Six patients were enrolled and received 4 to 8 autologous Vγ9Vδ2 T cell administrations every four weeks. The number of Vγ9Vδ2 T cells transferred ranged from 3.0 to 20.0 × 10^9^ cells. The treatment was safe and well tolerated and clinical efficacy was assessed by M-protein levels in the serum. In four patients, the M-protein level remained at baseline, and elevation was observed in two patients.

Wada *et al.* conducted a pilot study to evaluate the safety of weekly intraperitoneal injections of *in vitro* expanded Vγ9Vδ2 T cells together with Zol for the treatment of malignant ascites caused by peritoneal dissemination of gastric cancer [27]. PBMC were collected by leukapheresis, cryopreserved, and stored until use. PBMCs were stimulated by 5 μM of Zol and expanded in the presence of 1,000 IU/mL of IL-2 for 14 days. A total of four injections of Vγ9Vδ2 T cells were performed weekly. Seven patients received an intravenous injection of 1 mg of Zol and then three intraperitoneal injections to sensitize tumor cells one day before the transfer of 0.6 to 69.8 × 10^9^ Vγ9Vδ2 T cells. None of the patients experienced abdominal pain or any other toxicity when Vγ9Vδ2 T cells and Zol were intraperitoneally administered. Even though the patients were in an advanced stage (two were withdrawn from the study due to disease progression) and only three patients completed the fourth treatment, two patients experienced a decreased volume of ascites on CT and the number of tumor cells in ascites decreased gradually during the repeated injections of Vγ9Vδ2 T cells and Zol.

## 4. Discussion

We have reviewed the clinical trials of Vγ9Vδ2 T cell-based immunotherapies conducted over the last 16 years. The trials proved that Vγ9Vδ2 T cell-based immunotherapies were safe and well tolerated, and that the clinical benefits appeared to be mild to moderate. Although potent cytotoxic activity against various tumor cells has been confirmed *in vitro*, currently employed regimens have plenty of room for improvement.

Even in early-stage malignancy it was demonstrated that both the frequency and absolute number of Vγ9Vδ2 T cells were decreased in the peripheral blood in some patients [37]. It is generally difficult to expand Vγ9Vδ2 T cells efficiently with Zol plus IL-2 *in vitro* when the initial frequency of peripheral blood Vγ9Vδ2 T cells is relatively low. It is thus recommended that patients be examined for the frequency and absolute number of peripheral blood Vγ9Vδ2 T cells before enrollment in these studies [11]. Favorable clinical outcomes were related to higher frequency of peripheral blood Vγ9Vδ2 T cells and higher total number of *in vitro* cultured Vγ9Vδ2 T cells used for adoptive immunotherapy. In fact, six of thirteen patients with locally advanced RCC who showed increased frequency of peripheral blood Vγ9Vδ2 T cells over 9.6% of CD3^+^ cells before surgery survived more than ten years after the surgery, but among seven of thirteen patients whose peripheral blood Vγ9Vδ2 T cells were less than 6.8% of CD3^+^ cells before surgery, four died due to RCC [52]. Recent studies demonstrated that IL-18 enhanced the *in vitro* expansion of Vγ9Vδ2 T cells from patients, irrespective of the frequency of Vγ9Vδ2 T cells [35,37]. Although the precise mechanism has not yet been fully clarified, it is evident that IL-2 and IL-18 promote the maturation of helper NK cells expressing antigen-presenting cell (APC)-associated molecules such as CD86, HLA-DR, and HLA-DQ, which, in turn, enhance the expansion of Vγ9Vδ2 T cells. It is thus necessary to use IL-18 to expand Vγ9Vδ2 T cells for the enrollment of patients exhibiting low frequency of peripheral blood Vγ9Vδ2 T cells and/or low expansion rate in *in vitro* culture.

The adoptive transfer of Vγ9Vδ2 T cells efficiently supplies effector cells to patients. Vγ9Vδ2 T cells exhibit potent cytotoxic activity against tumor cells such as Daudi Burkitt’s lymphoma and RPMI8226 MM. When tumor cells are sensitized with N-bis, Vγ9Vδ2 T cells recognize the tumor cells in a Vγ9Vδ2 TCR-dependent manner and efficiently kill them. Based on this finding, infusion of Zol for the *in vivo* sensitization of tumor cells has been employed in Vγ9Vδ2 T cell-based immunotherapy. It should be, however, carefully examined whether or not the Zol infusion is beneficial for patients. It is easy to expand Vγ9Vδ2 T cells using phosphoantigens or Zol and IL-2 on a large scale [24,27,32]. When the patients were infused with Zol, however, the frequency and number of peripheral blood Vγ9Vδ2 T cells are markedly reduced and Vγ9Vδ2 T cells become anergic to both phosphoantigens and N-bis [14,23,24]. N-bis inhibits osteoclastic bone resorption by blocking FPPS, an enzyme in the mevalonate pathway. The use of intravenous N-bis is also associated with the appearance of APR, because the inhibition of FPPS by N-bis results in the intracellular accumulation of IPP, allowing Vγ9Vδ2 T cells to produce inflammatory cytokines such as IFN-γ and TNF-α. APR is in fact associated with the frequency and number of Vγ9Vδ2 T cells in peripheral blood. Over 25 Vγ9Vδ2 T cells/μL (*p* = 0.032) and >3.0% (*p* = 0.027) have been shown to be risk factors of APR [28]. APR occurs after the first injection of N-bis and no APR is generally observed after the second and further injections, possibly because of a reduction in the number of circulating Vγ9Vδ2 T cells [34]. Although the mechanism for the peripheral reduction of N-bis-reactive cells has not been fully investigated, activation-induced cell death (AICD) and/or incomplete activation of Vγ9Vδ2 T cells by unprofessional APCs appear to be involved in the exhaustion and anergy of Vγ9Vδ2 T cells. If AICD is the predominant mechanism, the administration of exogenous IL-2 might be helpful to expand and/or maintain the level of peripheral Vγ9Vδ2 T cells *in vivo*. Although several clinical trials involving Zol and IL-2 infusion have been designed and performed [11,13,14,16], the frequency and number of Vγ9Vδ2 T cells in the peripheral blood were reduced and the responses of Vγ9Vδ2 T cells to phosphoantigens and/or Zol were greatly impaired [14]. It is of note that the lower frequency of peripheral blood Vγ9Vδ2 T cells after Zol administration can be observed even one year after Zol administration [34]. In addition, patients who received N-bis via oral administration also showed significantly lower frequency and reduced number of peripheral blood Vγ9Vδ2 T cells [34]. It is thus important to exclude patients who have undergone N-bis administration, even via oral administration, in the selection of candidates for Vγ9Vδ2 T cell-based immunotherapies. In addition to N-bis, BrHPP, a pyrophosphomonoester antigen, might impair the responsiveness of Vγ9Vδ2 T cells after successive administration (every 3 weeks) [17]. Based on these findings, it is essential that clinical studies be conducted to explore a novel approach to stably expand and maintain the responsiveness and functions of Vγ9Vδ2 T cells.

A recent study demonstrated that IL-21 dramatically increased the cytotoxic activity of Vγ9Vδ2 T cells [38] and that IL-18 promoted the expansion of Vγ9Vδ2 T cells [35,37]. A combination of IL-18 and IL-21 has been used in clinical trials on NHL and melanoma; the regimens were well tolerated and provided significant clinical benefits [39,40]. The administration of Zol, IL-2, IL-21, and/or IL-18 might be promising combinations for the *in vivo* stimulation and expansion of Vγ9Vδ2 T cells. The therapeutic use of IL-18 and IL-21 must, however, be carefully and extensively examined and developed as translational research, because it is well known that the cytokines transduce signals related to cellular survival, proliferation, and differentiation and, therefore, may help tumor growth. It is also noteworthy that biological therapeutics may be used to enhance anti-tumor activity, because the cytotoxic activity of γδ T cells was significantly augmented by a conformational change in γδ TCR complexes induced by a certain monoclonal antibody against CD3 and the subsequent signal transduction through PI3K/Akt and Ras/Ark pathways [53].

The advantages of the adoptive transfer of autologous Vγ9Vδ2 T cells over the *in vivo* activation of Vγ9Vδ2 T cells are to be able (1) to avoid the exhaustion of Vγ9Vδ2 T cells and/or hypo-responsiveness to specific antigens; (2) to prevent the contact between Vγ9Vδ2 T cells and unprofessional APCs that may lead Vγ9Vδ2 T cells to anergy; 3) to easily obtain a large number of Vγ9Vδ2 T cells, 4) to manipulate Vγ9Vδ2 T cells *in vitro*, such as the augmentation of cytotoxicity using IL-18, IL-21, and other cytokines, the introduction of genes encoding receptors for tumor antigens, and the quality control of the effector cells; 4) to use allogeneic Vγ9Vδ2 T cells [54]; and 5) to administer Vγ9Vδ2 T cells locally, for example, to the intraperitoneal cavity, the enucleated cavity of the brain tumor, and the bladder.

There are several methods to stimulate and expand Vγ9Vδ2 T cells. Phosphoantigens or third-generation N-bis have been used as stimulants, and both classes of compounds yield a high level of stimulatory activity. Activation of Vγ9Vδ2 T cells by N-bis requires antigen presentation by monocyte-lineage cells [33]. Monocytes increase in the peripheral blood of patients with advanced cancer and the circulating monocytes actively infiltrate the tumor tissues, which might alter the tumor microenvironment, resulting in the acceleration of tumor progression. When these cells uptake N-bis, the function of Vγ9Vδ2 T cells in cancer patients might be influenced. In most of the studies of Vγ9Vδ2 T cell therapy, IL-2 was used for the expansion of Vγ9Vδ2 T cells, in which the concentration of IL-2 ranged from 100 to 1,000 IU/mL. In our experience, 100 IU/mL of IL-2 is appropriate to expand Vγ9Vδ2 T cells for adoptive immunotherapy, because too much expansion of Vγ9Vδ2 T cells by a higher concentration of IL-2 might reduce telomere length, resulting in apoptosis of Vγ9Vδ2 T cells *in vivo*. One of our prostate cancer patients who experienced biochemical failure after radical prostatectomy received four monthly Vγ9Vδ2 T cell transfers. In this patient, the frequency and absolute number of Vγ9Vδ2 T cells in the peripheral blood increased from 6.1 to 46.8% and 51 to 629 cells/μL. The high frequency of peripheral blood Vγ9Vδ2 T cells was sustained over 3 years, with the average frequency and absolute number of Vγ9Vδ2 T cells in the peripheral blood during this period being 25.9% and 192 cells/μL. It is of note that the PSA level of the patient was stable, even after the course of γδ T cell therapy, without any additional treatment such as hormone therapy, salvage radiation therapy, or chemotherapy.

It is also unclear whether or not the administration of exogenous IL-2 is required for the adoptive transfer of Vγ9Vδ2 T cells. There are two trials with or without exogenous IL-2 after the administration of *ex vivo* activated Vγ9Vδ2 T cells to patients with solid tumors [23,24]. CD25 was swiftly expressed in Vγ9Vδ2 T cells after stimulation with antigens. The CD25 expression declined after 10 to 11 days of culture to a baseline level at day 14. To sustain the functions of cultured Vγ9Vδ2 T cells *in vivo*, the administration of exogenous IL-2 may be useful as long as Vγ9Vδ2 T cells express CD25.

The administration of Vγ9Vδ2 T cells into a local cavity, not systemically, is another strategy. Wada and coworkers injected *ex vivo* cultured Vγ9Vδ2 T cells into the intraperitoneal cavity of patients with malignant ascites following Zol administration [27]. In this system, the exhaustion of Vγ9Vδ2 T cells by Zol might be avoided. Yuasa and coworkers reported that the intravesical administration of *in vitro* cultured Vγ9Vδ2 T cells prevented the growth of bladder cancer in a murine model [55]. Lamb and coworkers demonstrated that the administration of Vγ9Vδ2 T cells into the enucleated cavity of the brain tumor exhibited potent cytotoxic activity against GBM [56,57].

A combination of adoptive immunotherapy with immune checkpoint blockade is another approach to enhance the anti-tumor activity of immune effector cells, including Vγ9Vδ2 T cells. The recent clinical success of nivolumab, an anti-programmed death-1 (PD-1) mAb, and ipilimumab, an anti-cytotoxic T lymphocyte-associated protein-4 (CTLA-4) mAb, may bring about a paradigm shift in the treatment of patients with advanced cancer [58,59,60]. Phosphoantigen-stimulated Vγ9Vδ2 T cells express PD-1 [61] and blockade of the PD-1 and PD-L1 interaction enhances the cytotoxic activity of Vγ9Vδ2 T cells. Because there are many immune checkpoint molecules besides PD-1 and CTLA-4, such as LAG-3 and TIM-3, further studies are necessary to determine which combination is the most efficacious. A clinical trial of a combination therapy using anti-PD-1, anti-CD137, and tumor-infiltrating lymphocytes in patients with advanced melanoma is ongoing and the clinical outcomes of combination therapies using peptide plus nivolumab or peptide plus nivolumab and ipilimumab for advanced melanoma have recently been disclosed [62]. Further basic studies and clinical trials will give us additional clues on how to develop and establish successful immunotherapy approaches using Vγ9Vδ2 T cells.
